# Mathematical Modelling of Convective Drying of Orange By-Product and Its Influence on Phenolic Compounds and Ascorbic Acid Content, and Its Antioxidant Activity

**DOI:** 10.3390/foods12030500

**Published:** 2023-01-21

**Authors:** María del Carmen Razola-Díaz, Vito Verardo, Ana María Gómez-Caravaca, Belén García-Villanova, Eduardo Jesús Guerra-Hernández

**Affiliations:** 1 Department of Nutrition and Food Science, University of Granada, Campus of Cartuja, 18011 Granada, Spain; 2 Institute of Nutrition and Food Technology ‘José Matáix’, Biomedical Research Centre, University of Granada, Avda del Conocimiento sn, 18100 Granada, Spain; 3 Department of Analytical Chemistry, Faculty of Sciences, University of Granada, Avda Fuentenueva s/n, 18071 Granada, Spain

**Keywords:** HPLC-MS, orange peel, vitamin C, air-drying, polyphenols, hesperidin

## Abstract

Orange peel is one of the main by-products from juice processing, and is considered as a promising source of phenolic compounds with anti-carcinogenic, anti-inflammatory, anti-microbial and antioxidant properties. The drying is an essential step to ensure the storage of this by-product at an industrial level, in order to use it as a functional ingredient or as a nutraceutical. Thus, this research focuses on the evaluation of the effect of the convective air-drying process in orange by-products at three different temperatures (40, 60 and 80 °C) and air flows (0, 0.8 and 1.6 m/s) on the phenolic content (measured by HPLC-MS), the antioxidant activity (measured by DPPH, ABTS and FRAP), and the vitamin C content (measured by HPLC-UV/VIS). Moreover, the mathematical modelling of its drying kinetics was carried out to examine the orange by-product behavior. Among the tested mathematical models, the Page model reported the highest fit and the best drying conditions, which showed the lowest reductions were at 60 °C with an air flow of 1.6 m/s and taking 315 min.

## 1. Introduction

The orange is the most important citrus fruit produced in Spain, accounting for 3.3 million metric tons in 2019/2022, followed by tangerines (1.81 million metric tons), lemons (0.9 million metric tons) and grapefruits (0.06 million metric tons). Moreover, the volume produced in 2020 in Spain has increased by 0.3 million metric tons since 2012. Most of them are cultivated in the province of Andalusia, reaching 1.6 million tons in 2020. Although the home consumption of oranges was decreasing from 2013 to 2019, in 2020 there was a slight increase of 6.5% with respect to the previous year. It has been estimated that the per capita consumption of oranges in Spain is around 20 kg a year [1].

Fruit juice is a non-alcoholic beverage made from the extraction of the liquid contained in natural fruits such as oranges, pineapples, strawberries, cranberries, or mangoes. They are often consumed for their perceived nutritional benefits. For instance, orange juice has a perceived nutritional value because it contains vitamin C, folic acid and potassium. Although fruit juice consumption in the European Union used to be higher, the European Union has still historically consumed more fruit juice than any other region worldwide, accounting for a total of 9.1 million liters in 2018, making the region the biggest market for fruit juices and fruit nectars globally. Within this context, in 2021 Spain was the fourth producer of orange juice in the European Union after Germany, France and the United Kingdom, and the ninth worldwide. In fact, in Spanish orange juice accounts for 30% of the fruit juice and nectar sales-volume, followed by pineapple and peach. In 2017 the production of orange juice was 5.1 million liters [1].

However, from the process of production of orange juice there are generated residues which are often discarded as waste, which could be revalorized as a potential nutraceutical resource. The main by-product generated from the orange-juice industry is the peel, composed of the albedo, flavedo and rests of the pulp. It is rich in polyphenols, fiber and vitamins that have been attributed to antioxidant, antimicrobial, and cardioprotective activities among others. In general, agro-industrial waste has been given special attention for its potential use in developing high value-added products as nutraceuticals and at the same time reducing its environmental impact [2].

Drying is one of the most common and extended processes used to preserve the quality and stability and to extend the shelf-life of fruits, vegetables, and their by-products. It allows a reduction in the moisture content and water activity, avoiding spoilage during management and storage [3]. Orange by-product is a matrix characterized as having a high sugar and pectin content and at the same time high moisture (>50%), making it an excellent growth substrate for microorganisms. Thus, drying is necessary to ensure stability, providing long storage-life [4]. To find the best conditions for drying the orange by-product is an important matter, and some things should be taken into account. As previously reported [5], there are some authors that have studied the dehydration of the orange peel, comparing techniques for different purposes. Firstly, temperature is an important factor, because the bioactive and antioxidant compounds are thermolabiles, and high temperatures could lead to their destruction. On the other hand, from the industrial and economic point of view, the drying process should be short and low energy-consuming. Moreover, drying involves the simultaneous transference of heat and mass, turning it into a complex phenomenon. Therefore, the use of mathematical models to simulate its kinetics and explain the mechanism of the water transference during this operation is a useful tool to control the process [6]. Although some authors have evaluated the effect of drying in some parameters of the orange by-product [7,8] they did not discuss its drying kinetics. Recently, Deng et al. [9] analyzed the orange peel drying kinetics using only the Weibull model. Moreover, Afrin et al. [10] evaluated the orange-pomace drying kinetics through other models, but not focusing on the polyphenol content or antioxidant activity. In addition, in other fruits and vegetables the mathematical modelling of the drying process has been studied for the asparagus root [11], dragon fruit [12], edamame [13], pomelo albedo [14], uvaia fruit [15] or yam [16].There are few previous studies focusing on this aspect for orange by-products.

Considering all these facts, this research aims to evaluate the effect of the convective air-drying process in orange by-product on phenolic compounds, vitamin C and antioxidant activity in order to establish the best drying conditions that will permit a high-quality standard for this by-product that could be used as raw material for the production of functional ingredients for foods and/or nutraceuticals. Moreover, the mathematical modelling of its drying kinetics was carried out to examine the orange by-product behavior and to find the best model. To our knowledge, it is the first time that the modelling of the drying process of the orange by-product has been carried out at this range of temperatures (40–80 °C) and at three different air-flows (0–0.8–1.6 m/s) using five different mathematical models.

## 2. Materials and Methods

### 2.1. Chemicals and Samples

Ascorbic acid, Trolox, DPPH, ABTS and FRAP reagents were acquired from Sigma-Aldrich (St. Louis, MO, USA). Water was purified using a Milli-Q system (Millipore, Bedford, MA, USA). HPLC-grade water reagent and other reagents and solvents were purchased from Merck KGaA (Darmstadt, Germany).

Orange by-products (var. *Navelina*) were obtained after juice production. The resulting by-product was composed of the albedo, flavedo and the remains of the pulp of the orange with a moisture content of 70 ± 1.5%. The samples were dried, milled, and sieved to 100 µm, and kept at −18 °C until the analyses.

### 2.2. Drying Process and Kinetics

Samples of orange by-product were dried by convection drying using an oven (Memmert, Schwabach, Germany). The loss of weight was measured with a precision balance (Sartorius, Göttingen, Germany) for less than 15 s, until the moisture content was lower than 10% [9].

The temperatures tested were 40, 60 and 80 °C, in concordance to other authors [17,18,19,20,21,22]. In addition, three different air flows were tried, according to the oven possibilities: 0, 0.8 and 1.6 m/s.

At each time the moisture content was expressed as moisture ratio (MR,) according to its weight loss and remaining solid content; this is a dimensionless term calculated from Equation (1):

(1) $$\mathrm{MR}=\frac{M_{t}-M_{e}}{M_{0}-M_{e}}\;$$

where M_0_, M_t_ and M_e_ are the moisture content (g/g d.w.) at initial time, time t, and equilibrium, respectively.

The drying rate (DR) was calculated to express the moisture loss per unit of time, which can be calculated according to Equation (2):

(2) $$\mathrm{DR}=\frac{M_{t1}-M_{t2}}{t_{2}-t_{1}}$$

where M_t1_ and M_t2_ are the moisture content (g/g d.w.) at the time points t_1_ and t_2_, respectively.

The effective moisture diffusivity (D_eff_) was calculated according to Fick’s second law, assuming that the sample shrinkage is negligible, so the initial moisture distribution is uniform, and the moisture diffusivity is constant, as shown in Equation (3):

(3) $$\mathrm{MR}=\left(\frac{8}{\pi^{2}}\right)e^{\left(-\frac{\pi^{2}D_{eff}}{4L^{2}}\;t\right)}$$

where MR, D_eff_, L and t are the moisture ratio, effective moisture diffusivity, the half-thickness of the sample (m) and the time (min), respectively.

Additionally, the Arrhenius equation was used to calculate the activation energy (Ea) described in Equation (4):

(4) $$D_{eff}={\mathrm{Ae}}^{\left(-\mathrm{Ea}/\mathrm{RT}\right)}\;$$

where A, Ea, R and T are the pre-exponential factor of the Arrhenius equation, the activation energy (KJ/mol), the universal gas constant (8.314 J/mol K) and the temperature (K), respectively.

To predict accurately the drying kinetics of orange by-product is of huge importance in finding the model which fits the best to its drying behavior. Therefore, for the three different temperatures and the three different air flows, the experimental drying data was fitted with semi-theorical mathematical drying-models, presented in Table 1.

### 2.3. Statistical Parameters

Various statistical parameters were used to select the best mathematical model that predicts the drying kinetics of orange by-product, such as the coefficient of determination (R^2^), defined by Taylor [40]. Furthermore, other statistical parameters previously used by other authors [11,41] were also used to this purpose, such as square error (X^2^), the square error of estimation (SEE), root-mean-square error (RMSE), the mean absolute error (MAE) and the relative mean error (P_0_). Overall, these terms indicate how close the experimental and the prediction data are. The model is considered to have a high-quality fit, as the R^2^ value is closer to 1 and the X^2^, SEE, RMSE and MAE values are closer to 0. Moreover, the model is acceptable or with a good fit when P_0_ < 0.5 [6]. The named statistical parameters were calculated according to Equations (5)–(10), where the experimental and predicted dimensionless-moisture-ratios are expressed as MR_exp,i_ and MR_pred,i_, respectively. N refers to the number of observations and i to the number of constants in the model.

(5) $$R^{2}=\frac{\sum_{i=1}^{N}\left({\mathrm{MR}}_{\exp,i}-{\bar{\mathrm{MR}}}_{\mathrm{pred},i}\right)\times \left({\mathrm{MR}}_{\mathrm{pred},i}-{\bar{\mathrm{MR}}}_{\exp,i}\right)}{\sqrt{\sum_{i=1}^{N}{\left({\mathrm{MR}}_{\exp,i}-{\bar{\mathrm{MR}}}_{\mathrm{pred},i}\right)}^{2}\times \sum_{i=1}^{N}{\left({\mathrm{MR}}_{\exp,i}-{\bar{\mathrm{MR}}}_{\mathrm{pred},i}\right)}^{2}}}$$

(6) $$X^{2}=\frac{\sum_{i=1}^{N}{\left({\mathrm{MR}}_{\exp,i}-{\mathrm{MR}}_{\mathrm{pred},i}\right)}^{2}}{N-z}$$

(7) $$SEE=\sqrt{\frac{\sum_{i=1}^{N}{\left({\mathrm{MR}}_{\exp,i}-{\mathrm{MR}}_{\mathrm{pred},i}\right)}^{2}}{N-n_{i}}}$$

(8) $$RMSE={\left[\frac{1}{N}\sum_{i=1}^{N}{\left({\mathrm{MR}}_{\exp,i}-{\mathrm{MR}}_{\mathrm{pred},i}\right)}^{2}\right]}^{1/2}$$

(9) $$MAE=\frac{1}{N}\sum_{i=1}^{N}\left(MR_{pred,i}-MR_{exp,i}\right)$$

(10) $$P_{0}=\frac{1}{N}\sum_{i=1}^{N}\left|\frac{\left({\mathrm{MR}}_{\exp,i}-{\mathrm{MR}}_{\mathrm{pred},i}\right)}{{\mathrm{MR}}_{\exp,i}}\right|$$

### 2.4. Ultrasound Extraction of Phenolic Compounds

Briefly, 1 g of orange by-product powder was added to 15 mL solution of ethanol/water 80:20 *v*/*v*. The mixture was placed in an ultrasonic bath for 15 min and then it was centrifuged for 10 min at 9000 rpm. The supernatant was collected, and the extraction was repeated twice more. Finally, the supernatants were evaporated, and reconstituted in 1 mL of methanol/water (50:50, *v*/*v*). The final extracts were filtered with regenerated cellulose filters 0.2 μm (Millipore, Bedford, MA, USA) and stored at −18 °C until the analyses.

### 2.5. HPLC-MS Analysis of Phenolic Compounds

The analyses of the orange by-products dried under the different conditions were carried out in duplicate on an ACQUITY Ultra Performance LC system (Waters Corporation, Milford, MA, USA) coupled to an electrospray ionization (ESI) source operating in the negative mode, and a time-of-flight (TOF) mass detector (Waters Corporation, Milford, MA, USA). The compounds of interest were separated on an ACQUITY UPLC BEH Shield RP18 column (1.7 µm, 2.1 mm × 100 mm; Waters Corporation, Milford, MA, USA) at 40 °C, using a gradient previously stated by Verni et al. [42], using water containing 1% acetic acid as mobile phase A and acetonitrile as mobile phase B. Standard curves of chlorogenic acid, vanillic acid, ferulic acid, rutin, quercetin and catechin were performed, and the results are expressed as µg/g d.w. The data were elaborated using MassLynx 4.1 software (Waters Corporation, Milford, MA, USA).

### 2.6. HPLC-UV/VIS Analysis of Vitamin C

The determination of vitamin C was carried out using the method proposed by Mesías-García et al. [43]. To extract the ascorbic acid (AA), 0.5 mL of extract was mixed with 2.5 mL of a 10% (*w*/*v*) metaphosphoric acid solution and then diluted to a final volume of 25 mL in a glass volumetric flask with demineralized water. The mixture was homogenized and centrifuged at 9000 rpm for 15 min (room temperature). The supernatant was filtered through 0.20 μm Millex filters (Millipore, Bedford, MA, USA) and the samples were injected into the high-performance-liquid-chromatography (HPLC) system. To evaluate the total content of vitamin C (DHAA + AA) present in the samples, the reduction of DHAA to AA must be carried out. For this, a dithiothreitol (DTT) solution (1 mg/mL diluted in 45% K_2_HPO_4_) was prepared as a reducing agent, and an aliquot of 0.2 mL was added to 1 mL of the filtered sample, obtained in the AA analysis. The mixtures were kept in the dark for 30 min at room temperature, then the reduction was stopped by the addition of 0.2 mL of 2 M H_3_PO_4_ and the samples were injected into the HPLC system. DHAA content was calculated by the difference between the total vitamin C (after DHAA reduction) and initial AA (before reduction). Both determinations (AA and vitamin C) were performed in triplicate. AA and vitamin C were analyzed using HPLC with an UV/VIS detector at 25 °C. Separations were performed on a Gemini 5 μm C18 110 Å (150 × 4.6 mm) Phenomenex column. The measurement was carried out under isocratic conditions, using as the mobile phase demineralized water acidified with sulfuric acid at a pH of 2.2, flow rate of 0.6 mL/min, and a wavelength of 245 nm. The standard curve of AA (from 2.5 to 100 ppm) was created, and the results are expressed as µg AA/g d.w.

### 2.7. Antioxidant Assays

The antioxidant activity was evaluated in all the samples, using three different methods in duplicate. The DPPH assay was carried out according to a method proposed by several authors [44,45]. A total of 100 µL of each extract was added to 2.9 mL of DPPH, and after rapid stirring the bleaching power of the extract was observed in a time interval of 0 to 30 min, at 517 nm. The ABTS method was carried out according to Re et al. (1999) [46]. The monocation ABTS^•+^ was generated by oxidation of the ABTS with potassium persulfate in the dark at room temperature for 12–24 h. For each extract, 1 mL of the ABTS solution was added to 0.01 mL of extract and the detriment of absorbance was measured for 30 min at 734 nm. The FRAP assay was carried out following the procedure developed by Pulido et al. [47]. It is based in the reduction of Fe^3+^ to Fe^2+^ by the antioxidant substances. A total of 30 µL of each extract was added to 90 µL of distilled water and 900 µL of the FRAP reagent. It was kept for 30 min at 37 °C, and measured in the spectrophotometer at 595 nm. Standard curves of TE (1, 5, 10, 20, 50, 80, 100, 150, 200 ppm) were elaborated for each assay. Results are expressed as mg TE/g d.w.

## 3. Results and Discussion

### 3.1. Drying Kinetics and Modelling

**Figure 1:**
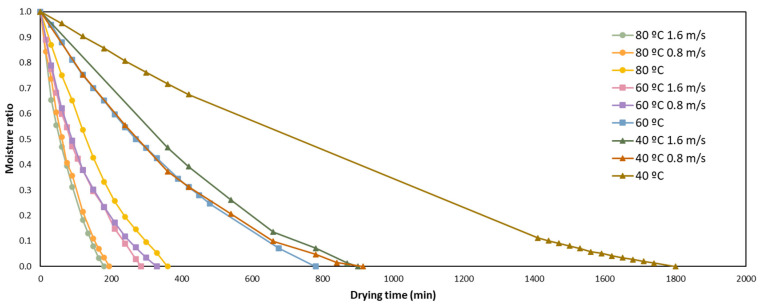
Drying-kinetics curves of orange peel under different temperatures and air flows.

The drying curve of orange by-product is shown in Figure 1; the changes of the moisture ratio of the orange peel through the drying time at different temperatures (40, 60 and 80 °C) and air flows (0, 0.8 and 1.2 m/s) are reported.

Without air flow, the drying time required to achieve the final moisture were 360, 720 and 1800 min, at 80, 60 and 40 °C, respectively. The temperature had a high influence on the drying of orange by-product. As expected, using higher temperature for less time was needed to reach the lower moisture-ratio. In the same way, the air flow was demonstrated to affect the drying. Using an air flow of 1.6 m/s, the time reduction was of 50% at all the temperatures tested, compared to not using air flow. There were not significative (*p* < 0.05) differences in time reduction with an air flow of 0.8 or 1.6 m/s. As a combination of temperature and air flow, drying at 80 °C with an air flow of 1.6 m/s was the quickest, needing only 180 min. However, to evaluate and select the best drying conditions, other parameters should be measured.

**Figure 2:**
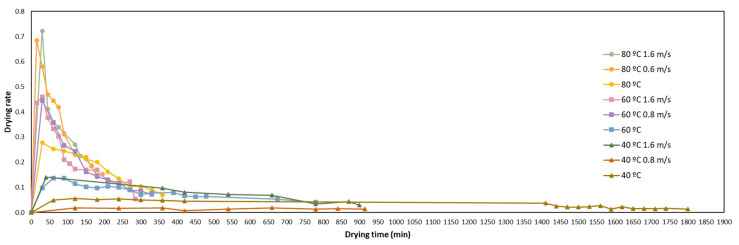
Drying-rate curves of orange peel under different temperatures and airflows.

Figure 2 shows the average drying rates of the orange peels dried at three different temperatures and airflows, against time. Depending on the drying conditions, the initial drying rate reached the maximum value at 30–45 min. The drying rate declined rapidly after this time. These facts can be due to the rapid evaporation of the surface moisture occurring in the first hour and, after that, to the evaporation of the internal moisture. This tendency has been previously reported by other authors in other matrices such as mentha [48], rosemary [49] and thyme [50]. The increase in temperature enhanced the drying rate considerably. In fact, without airflow, the drying rate increased 78.6% when drying at 80 °C, compared to drying at 40 °C. Furthermore, a consistent effect was found with the airflow. The highest impact on the drying rate with the airflow was at 80 °C (an increase of 61.1% when using 1.6 m/s of airflow). However, higher increases were also found with the airflow when drying at 60 °C (69.6%) and 40 °C (57.2%). Figure 2 shows that the samples dried at 80 °C using airflow reached the highest drying rate among all the tested conditions.

As regards the kinetics of drying, in Figure 1 it can be clearly appreciated that there are two stages of drying. The first one is the most rapid phase, and is majorly linear. In this step we appreciate the evaporation of the water of the superficial layers of the orange by-product. The second one is an exponential phase that usually takes longer, and is when the remaining water content diffuses to the surface. Therefore, the mathematical modelling of the drying curves is important for a better control of the drying process and better quality of the orange by-product.

To evaluate which model predicts the best drying kinetics of the orange by-product, the statistical parameters presented in Table 2 such as R^2^, X^2^, RMSE, P_0_ and MAE were taken into account. Overall, these terms indicate how the experimental and the prediction data are comparable. They were calculated at all the three temperatures at the three different air flows for the five proposed mathematical models.

The model is considered to have a high-quality fit, as the R^2^ value is closer to 1 and the X^2^, SEE, RMSE, P_0_ and MAE values are closer to 0. In general, all the models gave satisfactory results, with high coefficients of determination (R^2^ > 0.85). Among the five tested mathematical models when not using air flow and with an air flow of 0.8 m/s, the Page model fitted better at all the three temperatures, giving R^2^ values from 0.9784 to 0.9950. When using an air flow of 0.8, the best model adjustment changed, depending on the temperature. At 40 and 60 °C, the Page model reported the highest values of R^2^, keeping X^2^, SEE, RMSE and MAE the lowest. However, at 80 °C the Lewis model was found to have a better fitting with the experimental data. These results demonstrated that, depending on the temperature and air flow used when drying the orange by-product by convective drying, the drying kinetics vary. Additionally, the values obtained for all the constants of the five methods are exposed in Table 3, such as the drying-rate constants (k), the a for the Henderson and Pabis model, the N for the Page model, a and c for the logarithmic model, and a, b and n for the Weibull model. None of these parameters were constant, and exhibited temperature and air-flow dependence in concordance with other authors [14,41].

The effective moisture diffusivity (D_eff_) was calculated according to the Fick’s second law of diffusion for the three different drying temperatures and air flows for the five mathematical models tested. The obtained values ranged between 10^−5^ and 10^−9^ m^2^/s in concordance with the ranges reported by other authors [14,16,22,51,52,53].

The activation energy (Ea) is the required energy for the initialization of the moisture diffusion from inside to the outside of the sample. The activation energy and the pre-exponential factors for the five studied models at the three different air flows were calculated using the Arrhenius equation from the plot of ln(k) versus 1/T, and the results are exposed in Table 4. In all cases, the Ea was calculated in the temperature range from 313 to 353 K.

For the orange by-product dried with an air flow of 1.6 m/s, 0.8 m/s or without air flow, the Ea value ranges were 41.98–139.57, 31.16–60.60 and 30.68–55.82, respectively. Those results were in concordance with the data reported in other matrices [14,16,41]. In addition, Afrin et al. [10] reported for a modified Page model an activation energy of 53.07 kJ/mol when drying orange pomace without air flow in the range of 323–343 K, a value very similar to the one reported here. It is clear that with an air flow of 1.6 m/s, higher activation-energy is necessary, while without airflow, the Ea needed to start the moisture diffusion is lower. In all cases, the Page model gave the highest values of Ea. Comparing the five models, the Lewis and logarithmic models predicted the lowest energy of activation without air flow.

### 3.2. Effect of Drying on the Phenolic Content Measured by HPLC-MS

The effect of drying orange by-products on the phenolic content has been evaluated. Phenolic compounds were all identified and quantified according to previous research [54]. The results obtained for the phenolic compounds in the orange by-product treated at the three different temperatures and air-flows are reported in Table 5.

Regarding the phenolic acids, it can be appreciated that total feruloyl-isocitric acid and caffeoylmalic acid are at the highest concentration without air flow and at 40 °C, demonstrating them to be highly sensitive to high temperatures and oxygen exposure. In contrast, total feruloyl-galactaric-acid content increased when air flow was used. In general, at the three temperatures, the content of ferulic-acid derivatives was the highest at air flow 1.6 m/s and the lowest at air flow 0.8 m/s. In contrast, sinapic acid-O-glucuronide was higher at 0.8 m/s when drying the orange by-products at 40 and 80 °C. However, the highest concentration of this compound was found when drying at 60 °C with 1.6 m/s air flow. Therefore, the total content of phenolic acids was 3.5, 6.8 and 7.7% higher at 40, 60 and 80 °C, respectively, when drying at 1.6 m/s compared with without air flow. Pectolinarigenin is the major flavonoid found in these samples of orange by-product, and it is a flavone aglycone known for a series of biological properties including anti-inflammatory, antidiabetic and especially anticancer, against breast cancer [55]. Therefore, for pectolinaringenin and for total apigenin derivatives it has been seen that at 40 and 60 °C the content increased when increasing the air flow but at 80 °C it was the opposite, and the content decreased when increasing the air flow. It is due to the fact that at moderate temperature when introducing air, the time needed for drying is reduced, so the exposure to temperature is lower. However, in contrast, when increasing the temperature up to 80 °C and including air flow, although the time is even lower, the content degrades the most. A different tendency was appreciated for total rutin and total isorhamnetin-3-O-rutinoside. In this case the concentrations were higher in orange by-product treated without air circulation, demonstrating them to be more oxygen-sensitive. Regarding flavanones, the highest recovery was found when drying at 60 °C and 1.6 m/s of air flow and, in fact, the highest concentration of hesperidin, naringenin and naringin hydrate was achieved. Those compounds which have especially been extensively demonstrated to have several bioactivities in human health are those such as anti-cancer in lung and breast cancer [56,57,58], antidiabetic [59], anti-inflammatory [60], cardioprotective and anti-chloresterolemic [61,62,63,64,65,66,67,68], among others. In contrast, there were no significative changes in the content of hesperidin and narirutin at 80 °C, despite the different air flows. Regarding the total content of flavonoids, it was appreciated that there was a content of 6.2, 7.9 and 6.5% higher when drying at 40, 60 and 80 °C, respectively, at 1.6 m/s compared with without air flow. Therefore, in general, at all the temperatures tested it can be appreciated that the phenolic-compound content is higher when increasing the air flow, demonstrating that it has a huge impact on the phenolic-compound content. This is because when the air circulation is introduced, the time needed to achieve the final moisture is also reduced and, in consequence, the time of thermal exposure is lower. All these results are also statistically confirmed. A significant moderately strong negative-correlation has been found between the total phenolic acids, total flavonoids, and total phenolic compounds with temperature (r = −0.6222, −0.5219 and −0.6172, respectively). In contrast, a moderately strong positive-correlation of the phenolic compounds was found with the air flow being strongest, in the case of total flavonoids (r = 0.6679) and specifically total flavanones (r = 0.5832) and narirutin (r = 0.7190). There is a clear tendency of an increment in the total phenolic-compound content when increasing the air flow. However, although this tendency is still taking place when drying at 80 °C, there is a higher reduction in the phenolic content, mainly attributable to the case-hardening process previously reported by other authors in other matrices. In contrast to us, Chen et al. (2011) [69] reported achieving higher amounts of phenolic compounds such as naringin, hesperidin, kaempferol and rutin in a linear way, when increasing the temperature from 50 to 100 °C for drying orange-peel extracts. Despite this, the results obtained here for these compounds are in the same range of magnitude.

### 3.3. Effect of Drying on the Vitamin C Content

The content of ascorbic acid in the orange by-products dried at the three different temperatures and air flows was measured using HPLC-UV/VIS, and the results are shown in Table 6. Briefly, the high correlation between ascorbic-acid content and the total content of vitamin C (r = 0.9656), confirmed that AA was the main compound that constitutes vitamin C.

The highest recovery was obtained when orange by-products were dried at 40 °C and with an air flow of 1.6 m/s. Indeed, a significant (*p* < 0.05) negative, strong correlation between the content of vitamin C and the temperature (r = −0.6790) was detected. In contrast, a significative (*p* < 0.1) positive, moderate correlation was found between the total vitamin C and the air flow (r = 0.5215). Therefore, it is revealed that there is a lower reduction of the content of vitamin C when increasing the air flow, but at the lower temperature. The vitamin C content of the orange by-product, and especially the ascorbic acid as the major form, has been demonstrated to be highly sensitive to the exposure to temperature with the time that can be reduced by using air circulation being the factor that determines the avoidance of the biggest degradations. Within this context, there was a reduction of approximately 13.5% when drying at 80 °C, or 6% when drying at 60 °C compared with drying at 40 °C. Similar results were reported by Afrin et al. [10], who also had an ascorbic-acid reduction of 7.6% when drying orange pomace at 70 °C, compared with drying at 50 °C. This tendency has been also seen in other fruit and vegetables such as tomatoes, where a reduction of around 30% in the ascorbic acid content was reported when drying at 70 °C, compared with drying at 50 °C [70]. In addition, when a temperature of 60 °C with air flow of 1.6 m/s was applied, the highest ascorbic/dehydroascorbic ratio was observed, with no significative differences with the ratio observed at 40 °C and 0.8 m/s air flow. Through these trials, it can be confirmed that the oxygen exposure with the air flow of the samples during drying entails a higher oxidation of ascorbic acid to dehydroascorbic acid but no decrease in its total vitamin C content.

### 3.4. Effect of Drying on the Antioxidant Activity

**Figure 3:**
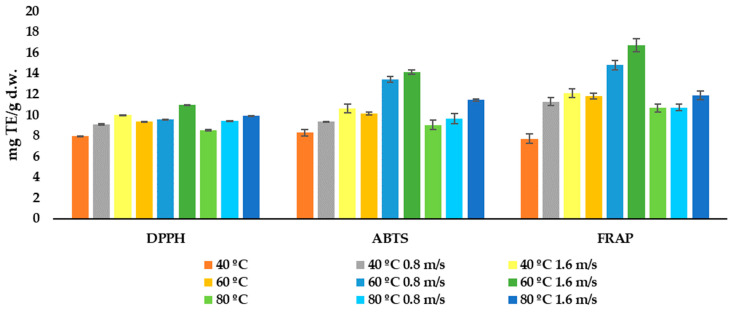
Antioxidant activity measured using DPPH, ABTS and FRAP of the orange by-product dried by convective drying at different temperatures and air flows. The results are expressed as mg TE (Trolox equivalents)/g d.w.

The antioxidant activity of the orange by-products dried at the three different temperatures and air flows was measured using three different methods, DPPH, ABTS and FRAP, and the results are shown in Figure 3. The result ranges were 7.97–10.99, 8.27–14.13 and 7.70–16.69 mg TE/g d.w. for the DPPH, ABTS and FRAP assays, respectively. All methods showed a significant (*p* < 0.05) strong, positive correlation between each other (DPPH vs. ABTS r = 0.8357; DPPH vs. FRAP r = 0.8643; ABTS vs. FRAP r = 0.9512).

All the three methods had a strong positive correlation (*p* < 0.05), with the total content of flavonoids being the strongest in the case of DPPH. Going further, this positive correlation is the highest for the total flavanones, and especially for hesperidin and narirutin. Moreover, when fixing a significance level of *p* < 0.1, a moderately strong, positive correlation between the total phenolic acid content and the antioxidant-activity results for ABTS and FRAP was found. For the three tested methods at the three temperatures, it can be appreciated that the highest antioxidant activity was obtained for the orange by-product dried with an air flow of 1.6 m/s. This is statistically confirmed by a correlation between the antioxidant activity measured by the three methods and the air flow (r > 0.5921). Moreover, a significant (*p* < 0.1) moderately negative correlation with time was found for the DPPH (r = −0.5958), ABTS (r = −0.5539) and FRAP (r = −0.6156) assays. These data agreed with those of Deng et al. [9], which stated that drying the orange peel at a temperature between 50 and 70 °C achieved the highest antioxidant recoveries, measured by DPPH and FRAP at 65 °C. In addition, Chen et al. [53] reported the highest radical scavenging activity when orange peel extracts were dried at 100 °C.

## 4. Conclusions

The drying step is essential for the storage of orange by-products at an industrial level. To this aim, the convective drying of the orange by-product was studied at three different temperatures (40, 60 and 80 °C) and air flows (0, 0.8 and 1.6 m/s). All these parameters had a direct effect on the time needed to achieve a final moisture-content lower than 10%. In addition, the five tested mathematical-models for fitting the drying kinetics of the orange by-product demonstrated satisfactory results. However, the Lewis and Page models gave better adjustment, reporting higher coefficient of regressions and lower squared and absolute errors for the major temperatures and air flows tested. The application of the Page equation will allow us to maximize the exploitation of orange by-products, reducing economic and energy costs. Moreover, to select the best drying conditions, the phenolic-compound content was evaluated by HPLC-MS, the antioxidant activity by DPPH, ABTS and FRAP and the vitamin C content by HPLC-UV/VIS. In all cases, both the temperature and the air flow were demonstrated to have a huge influence. When increasing the air flow, the time needed to reach the final moisture content is reduced, so the exposure to temperature is reduced and, in consequence, the phenol, ascorbic acid and antioxidant-compound reduction is lower. However, at 80 °C, the case-hardening phenomenon was increased when increasing the air flow, reducing the bioactive content. Regarding the vitamin C content, this was better with the lowest temperature tested. However, reaching a compromise among all the parameters evaluated, the drying conditions selected were 60 °C, with an air flow of 1.6 m/s that takes 315 min and allows a lower reduction of phenolic compounds and antioxidant compounds, especially flavanones such as hesperidin, narirutin and pectolinaringenin, keeping an acceptable ascorbic acid content. For future research, it would be interesting to evaluate the use of innovative extraction-techniques for increasing the extraction of phenolic compounds in this dry by-product, with improved management and storage at an industrial level for potential activities, and its application in functional foods and nutraceuticals.

## Figures and Tables

**Table 1:** Non-linear regression models for predicting moisture ratio of orange by-product samples.

Model Name	Model Equation	References
**Lewis**	$\mathrm{MR}=e^{\left(-\mathrm{kt}\right)}$	[23,24,25,26]
**Henderson and Pabis**	$\mathrm{MR}={\mathrm{ae}}^{\left(-\mathrm{kt}\right)}$	[27,28,29,30,31]
**Page**	$\mathrm{MR}=e^{\left(-{\mathrm{kt}}^{N}\right)}$	[32,33,34,35]
**Logarithmic**	$\mathrm{MR}={a\;e}^{-\mathrm{kt}}+c$	[36,37,38,39]
**Weibull**	$\mathrm{MR}=a-{b\;e}^{-{\mathrm{kt}}^{N}}$	[9]

MR: Moisture Ratio; t: time; a, b, c, N and k are the model constants.

**Table 2:** Statistical analysis of the selected mathematical models at three different temperatures and air flows.

Model	T (°C)	Air Flow 1.6 m/s						Air Flow 0.8 m/s						No Air Flow					
		R^2^	X^2^	SEE	RMSE	MAE	P_0_	R^2^	X^2^	SEE	RSME	MAE	P_0_	R^2^	X^2^	SEE	RSME	MAE	P_0_
Lewis	40	0.9224	0.0096	0.0979	0.1494	0.0721	0.5174	0.955	0.0072	0.0847	0.1696	0.0656	0.2042	0.9739	0.0100	0.0998	0.1463	0.0660	0.1696
	60	0.9624	0.0029	0.0537	0.1348	0.0476	0.9138	0.9829	0.0018	0.0421	0.0837	0.0365	0.1512	0.9677	0.0028	0.0529	0.1292	0.0507	0.1060
	80	0.9984	0.0850	0.2915	0.7517	0.2267	0.1423	0.9702	0.0052	0.0719	0.1552	0.0599	0.3093	0.9765	0.0054	0.0732	0.2581	0.0596	0.2993
Henderson and Pabis	40	0.8371	0.0897	0.2995	0.1750	0.1578	1.0087	0.9622	0.0087	0.0930	0.0692	0.0608	0.1670	0.9397	0.0150	0.1226	0.0630	0.0685	0.1349
	60	0.9289	0.0064	0.0798	0.0718	0.0613	0.9684	0.9611	0.0050	0.0707	0.0566	0.0480	0.1373	0.9468	0.0043	0.0657	0.0406	0.0527	0.1042
	80	0.9352	0.0855	0.2924	0.7337	0.2326	0.1472	0.9543	0.0106	0.1030	0.0717	0.0687	0.2004	0.9603	0.0090	0.0951	0.1820	0.0642	0.2412
Page	40	0.9682	0.0004	0.0207	0.0013	0.0174	0.9524	0.9784	0.0007	0.0256	0.0171	0.0213	0.1016	0.9906	0.0006	0.0237	0.0001	0.0181	0.0634
	60	0.9850	0.0009	0.0292	0.0075	0.0229	1.0105	0.9888	0.0005	0.0216	0.0030	0.0176	0.0877	0.995	0.0006	0.0246	0.0064	0.0158	0.0422
	80	0.9670	0.0052	0.0718	0.2194	0.0662	1.3791	0.9843	0.0008	0.0289	0.0109	0.0240	0.1291	0.9903	0.0007	0.0261	0.0265	0.0194	0.1662
Logarithmic	40	0.8959	0.2128	0.4613	1.2140	0.4292	6.7565	0.9178	0.1138	0.3373	1.0278	0.3250	0.3856	0.9426	0.0751	0.2741	1.0456	0.2585	0.4213
	60	0.9289	0.0464	0.2154	0.8036	0.2075	2.2445	0.9611	0.0537	0.2318	0.7804	0.2253	0.4121	0.9468	0.0329	0.1814	0.7208	0.1699	0.2477
	80	0.9194	0.0212	0.1458	0.3491	0.1072	2.0326	0.9543	0.0608	0.2465	0.8178	0.2361	0.4897	0.9644	0.0895	0.2991	1.1845	0.2900	0.5417
Weibull	40	0.8969	1.0743	1.0365	2.9293	1.0357	14.6110	0.9719	0.7685	0.8766	2.7529	0.8705	0.5651	0.8895	0.6061	0.7785	2.1986	0.7182	0.5254
	60	0.9834	0.5099	0.7140	2.6252	0.6778	5.2870	0.9918	0.6706	0.8189	2.7735	0.8006	0.6357	0.9899	0.3268	0.5717	2.1481	0.5063	0.4372
	80	0.9651	0.7082	0.8415	2.7550	0.8307	6.6091	0.9831	0.5480	0.7403	2.4149	0.6971	0.6567	0.9958	0.4631	0.6805	3.2910	0.6098	0.6493

T: Temperature.

**Table 3:** Coefficients of Lewis, Henderson and Pabis, Page, logarithmic and Weibull models.

Air Flow	T (°C)	Model											
		Lewis	Henderson and Pabis		Page		Logarithmic			Weibull			
		k	k	a	k	N	k	a	c	k	a	b	n
1.6 m/s	40	0.004	0.005	1.824	0.000	1.850	0.005	1.368	0.456	0.000	1.000	−0.933	1.850
	60	0.010	0.011	1.212	0.006	1.091	0.011	0.909	0.303	0.006	1.007	−0.259	1.091
	80	0.057	0.058	1.062	0.005	1.179	0.019	0.742	0.247	0.007	1.001	−0.552	1.179
0.8 m/s	40	0.004	0.004	1.267	0.000	1.329	0.005	1.138	0.379	0.001	1.001	−0.691	1.329
	60	0.009	0.010	1.204	0.005	1.107	0.010	0.903	0.301	0.005	1.006	−0.438	1.107
	80	0.015	0.017	1.290	0.006	1.187	0.017	0.967	0.322	0.005	1.007	−0.234	1.187
No	40	0.002	0.002	1.408	0.000	1.343	0.002	0.998	0.333	0.000	1.000	−0.229	1.343
	60	0.003	0.004	1.181	0.001	1.188	0.004	0.886	0.295	0.001	1.001	−0.062	1.188
	80	0.007	0.008	1.284	0.002	1.263	0.009	1.069	0.356	0.002	1.002	−0.042	1.263

T: Temperature.

**Table 4:** Estimated energy of activation (Ea) and pre-exponential factor (A) for orange peels dried with different air flows in the temperature range 313–353 K.

Model	Air Flow 1.6 m/s		Air Flow 0.8 m/s		No Air Flow	
	Ea (kJ/mol)	A	Ea (kJ/mol)	A	Ea (kJ/mol)	A
Lewis	63.49	30,576.90	32.14	4.26	30.68	17.10
Henderson and Pabis	58.37	5450.70	35.12	1.35	31.63	10.61
Page	139.57	1.62 × 10^15^	60.60	1972.78	55.82	92.13
Logarithmic	33.32	2.31	31.16	5.34	32.68	7.22
Weibull	41.98	6.66	55.67	351.53	49.38	9.54

**Table 5:** Phenolic compounds in orange by-product dried at three different temperatures and air flows expressed as µg/g d.w. with the average ± standard deviation.

	40 °C			60 °C			80 °C		
	No Air Flow	Air Flow 0.8 m/s	Air Flow 1.6 m/s	No Air Flow	Air Flow 0.8 m/s	Air Flow 1.6 m/s	No Air Flow	Air Flow 0.8 m/s	Air Flow 1.6 m/s
Total phenolic compounds	5456.16 ± 26.04	5527.02 ± 25.73	5682.05 ± 24.84	5629.07 ± 20.61	5685.66 ± 25.27	6051.14 ± 29.42	4819.98 ± 21.81	5009.58 ± 28.24	5209.04 ± 20.51
Total phenolic acids	4455.51 ± 12.66	4468.87 ± 12.06	4614.93 ± 10.75	4606.92 ± 7.04	4644.09 ± 11.63	4941.70 ± 15.87	3864.45 ± 8.63	4032.31 ± 15.04	4186.94 ± 7.57
Feruloyl galactaric acid isomer a	2404.84 ± 1.36	2509.04 ± 3.68	2683.77 ± 6.18	2674.52 ± 0.65	2740.43 ± 1.55	2664.82 ± 2.67	2296.02 ± 2.61	2180.15 ± 4.10	2376.07 ± 1.39
Feruloyl galactaric acid isomer b	1017.20 ± 3.48	1062.22 ± 1.59	1112.53 ± 1.78	1096.90 ± 1.10	994.47 ± 4.83	1158.10 ± 5.79	907.51 ± 1.77	923.03 ± 0.55	943.40 ± 0.96
Sinapinic acid-O-glucuronide	632.81 ± 4.69	660.80 ± 0.99	609.71 ± 1.81	583.90 ± 1.82	664.99 ± 3.82	739.48 ± 2.63	373.37 ± 2.59	586.58 ± 7.54	533.36 ± 2.51
Feruloyl isocitric acid isomer a	148.99 ± 1.64	107.74 ± 1.46	93.88 ± 0.49	122.21 ± 2.00	110.17 ± 0.57	150.14 ± 1.41	82.42 ± 1.20	101.45 ± 0.67	114.19 ± 0.60
Feruloyl isocitric acid isomer b	67.76 ± 0.75	28.36 ± 1.39	24.14 ± 0.15	21.07 ± 0.22	31.18 ± 0.48	51.66 ± 0.72	50.10 ± 0.03	66.44 ± 0.58	51.94 ± 0.68
Feruloyl isocitric acid isomer c	147.59 ± 0.70	77.32 ± 2.48	69.85 ± 0.26	85.65 ± 1.12	78.88 ± 0.32	142.24 ± 2.24	118.75 ± 0.42	151.24 ± 1.15	132.76 ± 0.87
Caffeoylmalic acid	36.32 ± 0.05	23.40 ± 0.46	21.04 ± 0.08	22.66 ± 0.11	23.97 ± 0.06	35.25 ± 0.40	36.27 ± 0.02	23.41 ± 0.45	35.22 ± 0.56
Total flavonoids	1000.65 ± 13.38	1058.15 ± 13.67	1067.12 ± 14.09	1022.15 ± 13.57	1041.57 ± 13.64	1109.43 ± 13.55	955.53 ± 12.95	977.27 ± 13.20	1022.09 ± 13.19
Isorhamnetin-3-O-rutinoside isomer a	18.55 ± 0.52	17.67 ± 0.50	16.35 ± 0.46	18.10 ± 0.50	18.15 ± 0.49	19.61 ± 0.53	15.96 ± 0.44	16.86 ± 0.48	16.22 ± 0.46
Isorhamnetin-3-O-rutinoside isomer b	3.88 ± 0.14	4.10 ± 0.15	3.87 ± 0.14	4.21 ± 0.15	4.02 ± 0.15	4.09 ± 0.14	3.29 ± 0.13	3.38 ± 0.13	4.02 ± 0.14
Isorhamnetin-3-O-rutinoside isomer c	4.39 ± 0.16	3.15 ± 0.12	4.33 ± 0.16	4.79 ± 0.17	4.19 ± 0.15	3.66 ± 0.13	4.07 ± 0.15	4.33 ± 0.16	5.47 ± 0.18
Prunin	18.71 ± 0.55	24.36 ± 0.68	23.75 ± 0.69	22.82 ± 0.66	11.75 ± 0.36	24.17 ± 0.64	18.81 ± 0.55	20.58 ± 0.60	18.79 ± 0.52
Didymin	9.87 ± 0.31	10.32 ± 0.31	10.21 ± 0.32	9.15 ± 0.29	11.82 ± 0.36	10.45 ± 0.30	9.60 ± 0.30	10.87 ± 0.33	9.74 ± 0.29
Pectolinarigenin	644.28 ± 4.60	675.57 ± 4.66	687.33 ± 4.88	667.32 ± 4.76	678.64 ± 4.86	715.95 ± 4.66	604.36 ± 4.35	615.58 ± 4.43	639.10 ± 4.34
Hesperidin	56.65 ± 1.07	58.25 ± 1.06	68.40 ± 1.26	66.58 ± 1.23	71.55 ± 1.32	71.99 ± 1.22	57.38 ± 1.08	57.23 ± 1.08	56.01 ± 1.01
Narirutin	40.17 ± 0.80	47.79 ± 0.90	49.02 ± 0.94	42.80 ± 0.85	47.16 ± 0.92	47.19 ± 0.85	46.93 ± 0.91	45.01 ± 0.88	45.21 ± 0.84
Naringenin	2.59 ± 0.11	0.96 ± 0.06	0.32 ± 0.05	1.99 ± 0.09	0.54 ± 0.05	3.48 ± 0.13	0.67 ± 0.06	0.61 ± 0.05	2.44 ± 0.10
Naringin hydrate	10.41 ± 0.32	13.54 ± 0.39	12.23 ± 0.37	11.43 ± 0.35	12.71 ± 0.39	13.98 ± 0.39	11.71 ± 0.36	11.05 ± 0.34	12.86 ± 0.37
Rutin isomer a	15.18 ± 0.45	16.22 ± 0.47	14.83 ± 0.44	15.04 ± 0.45	14.92 ± 0.45	15.41 ± 0.42	13.95 ± 0.42	15.86 ± 0.47	15.67 ± 0.44
Rutin isomer b	11.97 ± 0.36	10.53 ± 0.32	10.72 ± 0.33	10.91 ± 0.34	8.19 ± 0.26	8.32 ± 0.25	9.80 ± 0.31	11.18 ± 0.34	11.96 ± 0.35
3’,4’-Didemethylnobiletin	2.76 ± 0.12	2.20 ± 0.10	1.85 ± 0.09	0.80 ± 0.06	0.10 ± 0.04	2.79 ± 0.11	1.24 ± 0.07	0.48 ± 0.05	0.61 ± 0.05
Quercetin-3-O-rutinoside-7-O-Glucoside	1.95 ± 0.09	2.29 ± 0.10	2.45 ± 0.10	2.34 ± 0.10	2.84 ± 0.12	2.41 ± 0.10	2.59 ± 0.11	2.42 ± 0.10	2.32 ± 0.10
Apigenin 6-C-glucoside 8-C-arabinoside/Vitexin-O-pentoside isomer a	10.46 ± 0.32	10.80 ± 0.32	11.63 ± 0.36	9.88 ± 0.31	11.11 ± 0.34	12.23 ± 0.34	10.12 ± 0.31	9.94 ± 0.31	11.63 ± 0.34
Apigenin 6-C-glucoside 8-C-arabinoside/Vitexin-O-pentoside isomer b	13.04 ± 0.39	12.60 ± 0.37	13.08 ± 0.39	11.77 ± 0.36	12.62 ± 0.38	14.47 ± 0.40	12.28 ± 0.37	11.46 ± 0.35	14.33 ± 0.41
Apigenin-di-C-hexoside (Vicenin-2)	63.75 ± 1.18	70.19 ± 1.25	71.60 ± 1.31	66.79 ± 1.24	69.71 ± 1.29	78.36 ± 1.31	60.91 ± 1.14	65.73 ± 1.22	71.78 ± 1.25
Apigenin 7-O-neohesperidoside	4.54 ± 0.16	5.97 ± 0.20	5.27 ± 0.18	3.75 ± 0.14	5.28 ± 0.18	4.96 ± 0.16	4.77 ± 0.17	5.42 ± 0.19	6.24 ± 0.20
Kaempferol 3-apiosyl-(1->2)-galactoside/Luteolin-C-hexoside-C-pentoside isomer a	3.02 ± 0.12	3.86 ± 0.14	2.45 ± 0.11	3.04 ± 0.12	2.32 ± 0.10	3.30 ± 0.12	2.59 ± 0.11	2.38 ± 0.10	2.74 ± 0.11
Kaempferol 3-apiosyl-(1->2)-galactoside/Luteolin-C-hexoside-C-pentoside isomer b	<LOQ	<LOQ	2.58 ± 0.11	4.52 ± 0.16	1.94 ± 0.09	4.57 ± 0.15	1.53 ± 0.08	1.29 ± 0.07	0.03 ± 0.04
Kaempferol 3-[2″-glucosyl-6″-acetyl-galactoside] 7-glucoside isomer a	5.09 ± 0.18	3.69 ± 0.14	4.55 ± 0.16	3.31 ± 0.13	2.42 ± 0.10	3.30 ± 0.12	3.93 ± 0.15	2.34 ± 0.10	5.02 ± 0.17
Kaempferol 3-[2″-glucosyl-6″-acetyl-galactoside] 7-glucoside isomer b	5.17 ± 0.18	4.39 ± 0.15	2.90 ± 0.12	4.42 ± 0.16	3.39 ± 0.13	4.82 ± 0.16	4.84 ± 0.17	3.27 ± 0.13	5.60 ± 0.18
Kaempferol-dihexosyl acetate	50.66 ± 0.97	58.54 ± 1.07	43.42 ± 0.85	31.70 ± 0.66	43.49 ± 0.86	34.87 ± 0.66	51.92 ± 0.99	58.51 ± 1.10	61.64 ± 1.09
kaempferol 3-O-[3″,6″-di-O-(E)-cinnamoyl]-beta-D-glucopyranoside	<LOQ	<LOQ	0.99 ± 0.07	1.51 ± 0.08	0.78 ± 0.06	1.24 ± 0.07	0.06 ± 0.04	0.36 ± 0.05	<LOQ
Kaempferol 3-O-(6″-O-acetyl)glucoside-7-O-rhamnoside	0.63 ± 0.06	<LOQ	0.17 ± 0.04	<LOQ	<LOQ	<LOQ	<LOQ	<LOQ	0.11 ± 0.04
Kaempferol 3-O-sinapoyl-caffeoyl-sophoroside 7-O-glucoside isomer a	1.84 ± 0.09	1.30 ± 0.07	1.11 ± 0.07	1.26 ± 0.07	1.20 ± 0.07	1.99 ± 0.09	0.97 ± 0.06	0.78 ± 0.06	0.76 ± 0.06
Kaempferol 3-O-sinapoyl-caffeoyl-sophoroside 7-O-glucoside isomer b	0.78 ± 0.06	0.46 ± 0.05	0.73 ± 0.06	0.56 ± 0.05	0.15 ± 0.04	0.89 ± 0.06	0.33 ± 0.05	<LOQ	0.67 ± 0.06
Kaempferol 3-apiosyl-(1->4)-rhamnoside-7-rhamnoside	1.11 ± 0.07	0.84 ± 0.06	0.98 ± 0.06	1.37 ± 0.08	0.55 ± 0.05	0.94 ± 0.06	0.93 ± 0.06	0.36 ± 0.05	1.13 ± 0.07

**Table 6:** Vitamin C content in orange by-product dried at three different temperatures and air flows expressed as µg AA/g d.w. with average ± standard deviation.

	Ascorbic Acid	Dehydroascorbic Acid	Ascorbic Acid/Dehydroascorbic Acid Ratio	Total Vitamin C
40 °C	522.41 ± 0.00	435.51 ± 0.00	1.20	957.92 ± 0.00
40 °C 0.8 m/s	1007.67 ± 3.05	457.49 ± 1.39	2.20	1465.17 ± 4.44
40 °C 1.6 m/s	1029.62 ± 5.38	503.60 ± 2.63	2.04	1533.21 ± 8.00
60 °C	539.26 ± 0.04	342.30 ± 0.02	1.58	881.57 ± 0.06
60 °C 0.8 m/s	707.22 ± 1.81	339.75 ± 0.87	2.08	1046.97 ± 2.68
60 °C 1.6 m/s	715.01 ± 2.25	323.58 ± 1.02	2.21	1038.58 ± 3.27
80 °C	478.65 ± 2.21	349.71 ± 1.62	1.37	828.36 ± 3.83
80 °C 0.8 m/s	615.38 ± 3.85	347.15 ± 2.17	1.77	962.53 ± 6.02
80 °C 1.6 m/s	507.13 ± 3.52	487.84 ± 3.39	1.04	994.96 ± 6.91

## Data Availability

All related data and methods are presented in this paper.
